# Supplementary material for: Cullin3-KLHL15 ubiquitin ligase mediates CtIP protein turnover to fine-tune DNA-end resection
Source: Nat Commun. 2016 Aug 26;7:12628. doi: 10.1038/ncomms12628 (PMC5007465; doi:10.1038/ncomms12628)
Supplement: Supplementary Information — Supplementary Figures 1-9, Supplementary Table 1 and Supplementary References [file ncomms12628-s1.pdf]

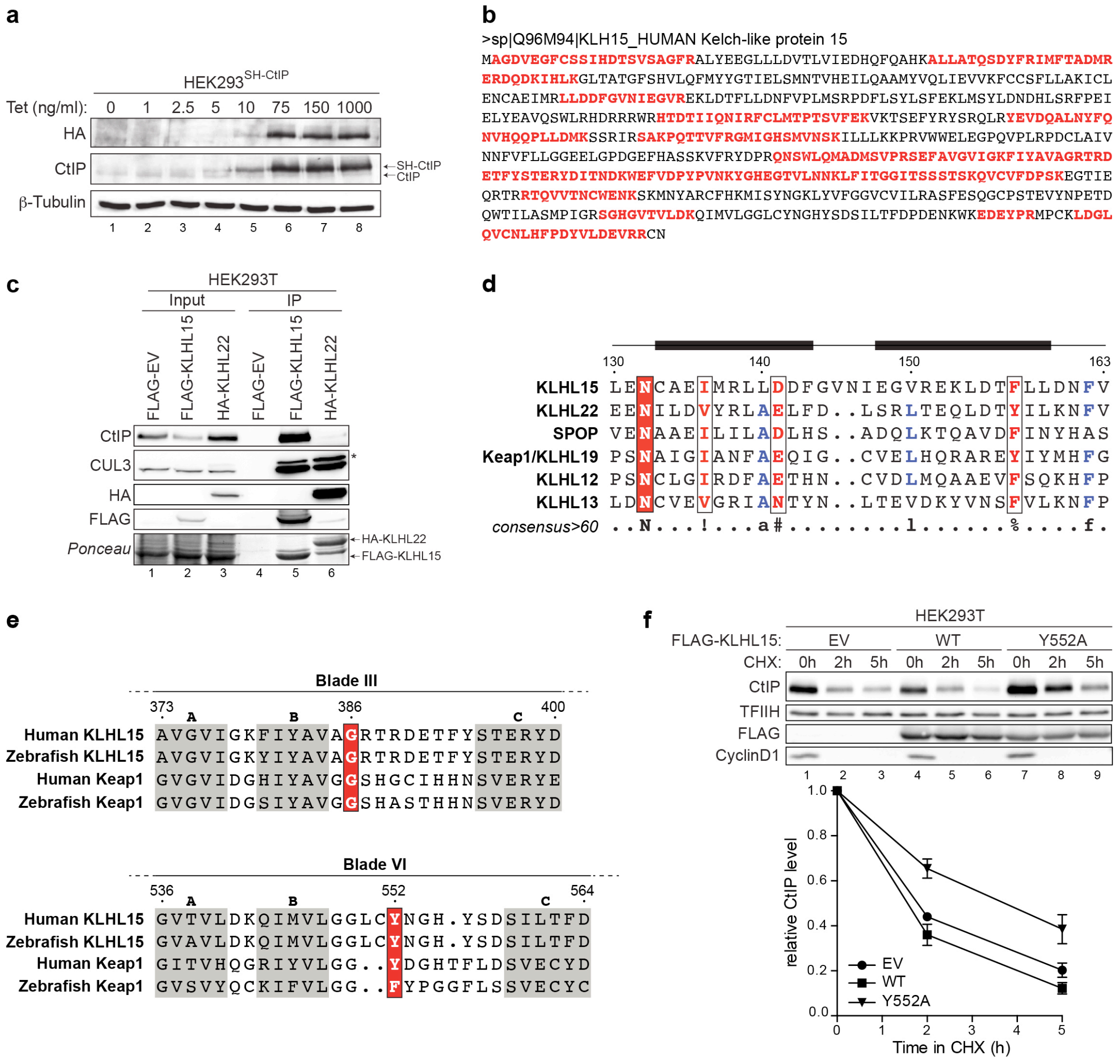

Supplementary Figure 1. Identification of human KLHL15, a Cullin-3 substrate adaptor, as a novel CtIP-interacting protein. a, HEK293 Flp-In T-REx cells stably expressing tetracycline (Tet)-inducible StrepHA-tagged CtIP (HEK293$^{SH\text{-}CtIP}$) were cultivated in presence of the indicated dose of Tet for 48 h. Whole- cell lysates were immunoblotted using the indicated antibodies. **b**, Amino acid (aa) sequence of human KLHL15 (604 aa). Tryptic peptides, corresponding to KLHL15 that were identified by mass spectrometry following tandem affinity purification of the CtIP bait fusion protein expressed in HEK293 cells, are depicted in bold red. **c**, HEK293T cells were transfected with either empty vector (EV), FLAG-KLHL15 or HA-KLHL22 expression constructs. 48 h after transfection, cells were lysed and whole-cell extracts were subjected to immunoprecipitation (IP) using either anti-FLAG M2 or anti-HA agarose resin. Inputs and recovered protein complexes were analysed by immunoblotting. **d**, Sequence alignment (*MultAlin*) of 32-34 aa corresponding to 3-boxes from five CUL3- interacting BTB proteins[1]. KLHL15 3-box residue numbers are shown on top. The paired alpha helices are indicated with black rectangles above the alignment. The highly conserved asparagine (N132 in KLHL15) is highlighted in a red box. **e**, Sequence alignments of regions corresponding to the third (upper panel) or the sixth (lower panel) kelch-repeat in KLHL15 and Keap1 from human and zebrafish. Three of the four β-strands (A–C) forming one 'blade' of the β-propeller structure are highlighted in gray[2]. Human KLHL15 residue numbers are shown on top. Highly conserved amino acid residues, G386 and Y552, are highlighted in a red box. **f**, HEK293T cells transfected with either empty vector (EV) or the indicated FLAG-KLHL15 expression constructs for 48 h were treated with cycloheximide (CHX, 100 μg ml^{-1}) for the indicated time points and analysed by immunoblotting (upper panel). Relative CtIP protein levels were determined by quantification of CtIP band intensity (normalized to TFIIH) with the ImageJ software (lower panel). Data are presented as mean values of densitometric quantification ± SEM (n =5).

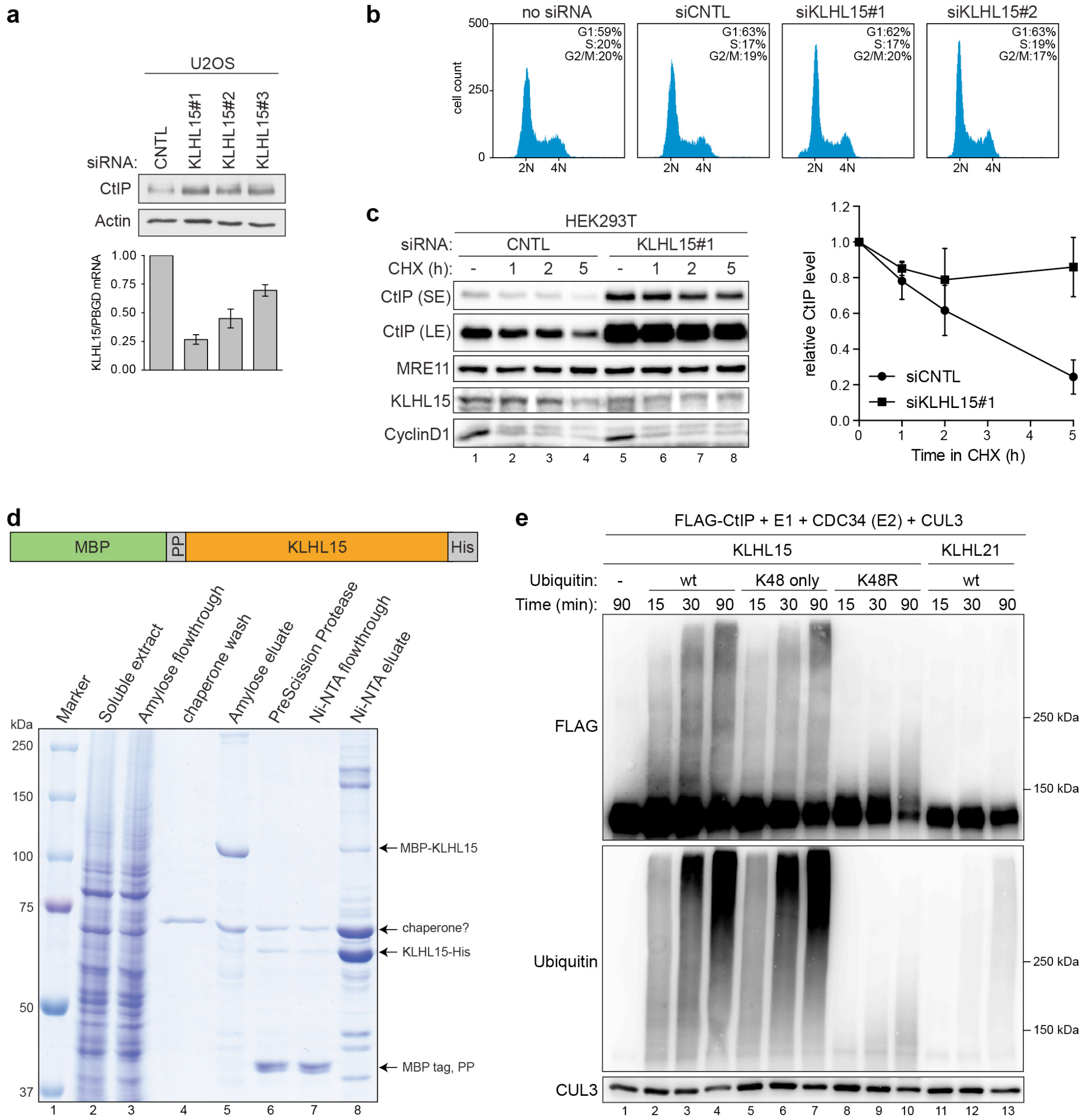

Supplementary Figure 2. KLHL15 decreases CtIP protein half-life by promoting its degradative K48-linked ubiquitination. **a**, U2OS cells transfected with the indicated siRNAs (20 nM) for 48 h were either analysed by immunoblotting (upper panel) or processed for RNA extraction followed by quantitative RT-PCR (lower panel). Relative KLHL15 mRNA levels are presented as the mean ± SD (n=3). **b,** FACS analysis of U2OS cells transfected with the indicated siRNAs (20 nM) for 48 h. **c,** U2OS cells were transfected with the indicated siRNA oligos. After 48 h, cells were treated with cycloheximide (CHX, 100 µg ml^{-1}) for the indicated time points and whole cell lysates were analysed by immunoblotting (left panel). SE and LE; short and long exposure times of the same immunoblot. Relative CtIP protein levels were determined by quantification of CtIP band intensity (normalized to MRE11) with the ImageJ software (right panel). Data are presented as mean values of densitometric quantification ± SEM (n =3). **d**, KLHL15 purification from Sf9 insect cells showing fractions analysed by SDS-PAGE. The mass of molecular weight markers is indicated on the left, and the positions of the respective recombinant constructs are indicated on the right. The gel was photographed upon staining with Coomassie Brilliant Blue. MBP, Maltose-binding protein. PP, PreScisson Protease cleavage site. **e,** FLAG-CtIP expressed and purified from HEK293T cells was incubated with ATP, E1, Cdc34, CUL3, KLHL15 or KLHL21 in the presence of different ubiquitin variants for the indicated time points at 37°C. Unmodified and ubiquitinated CtIP protein species were detected by immunoblotting with anti-FLAG antibody. The same immunoblot was stripped and reprobed with anti-ubiquitin antibody.

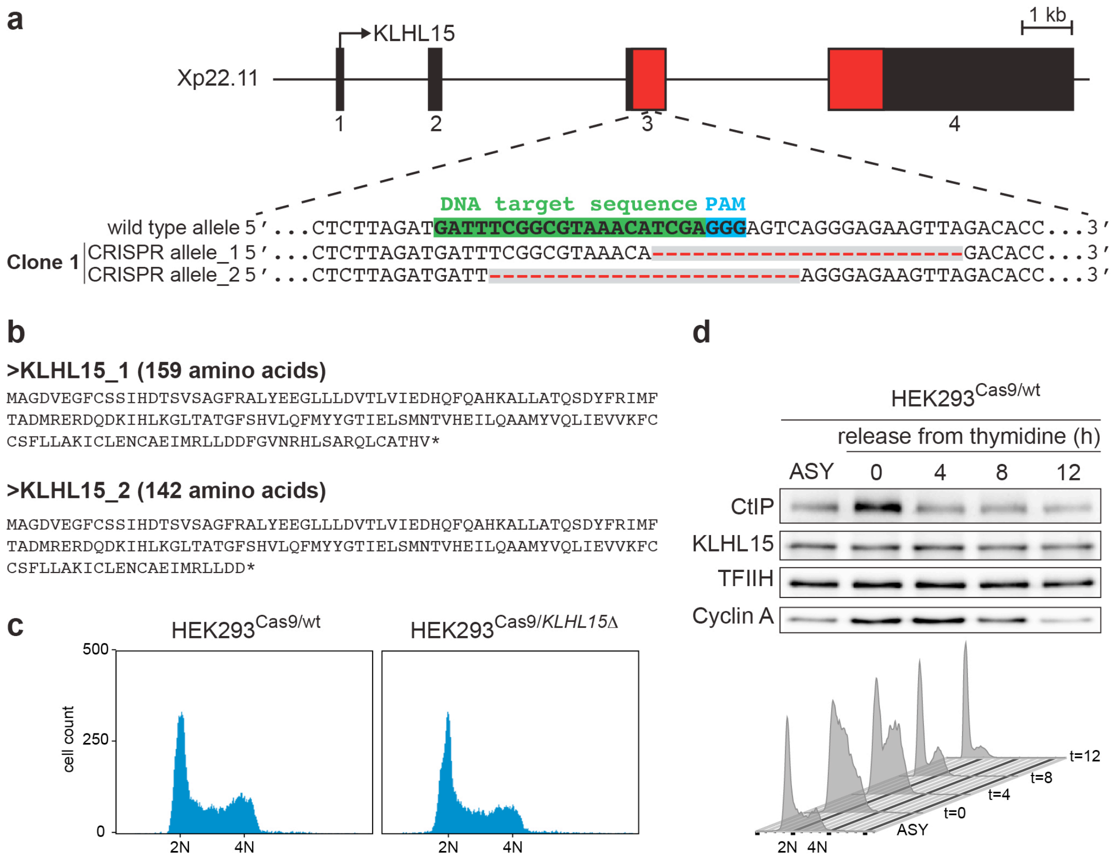

Supplementary Figure 3. Generation and characterization of HEK293$^{Cas9/KLHL15\Delta}$ cells. **a**, Schematic representation of human KLHL15 gene locus on chromosome Xp22.11. Exons are represented as black boxes and the protein coding region is indicated in red. The 20 nt sgRNA target sequence in Exon 3 of the KLHL15 wild-type (WT) gene is highlighted and green and the protospacer-adjcent motif (PAM) sequence is colored in blue. Allelic deletions introduced by CRISPR–Cas9 and identified from the PCR fragments generated from the HEK293$^{Cas9/KLHL15\Delta}$ clone 1 are indicated below. **b**, Predicted translation of the Open Reading Frames corresponding to mutated KLHL15 alleles identified in **a**. **c**, FACS analysis of HEK293$^{Cas9/wt}$ and HEK293$^{Cas9/KLHL15\Delta}$ cells. **d,** HEK293$^{Cas9/wt}$ cells were either grown asynchronously (ASY) or were synchronized using a single thymidine block with 2 mM thymidine for 16 h. At the indicated time points after release, cells were harvested and either lysed for western blot analysis (upper panel) or stained with DAPI and analyzed by flow cytometry (lower panel).

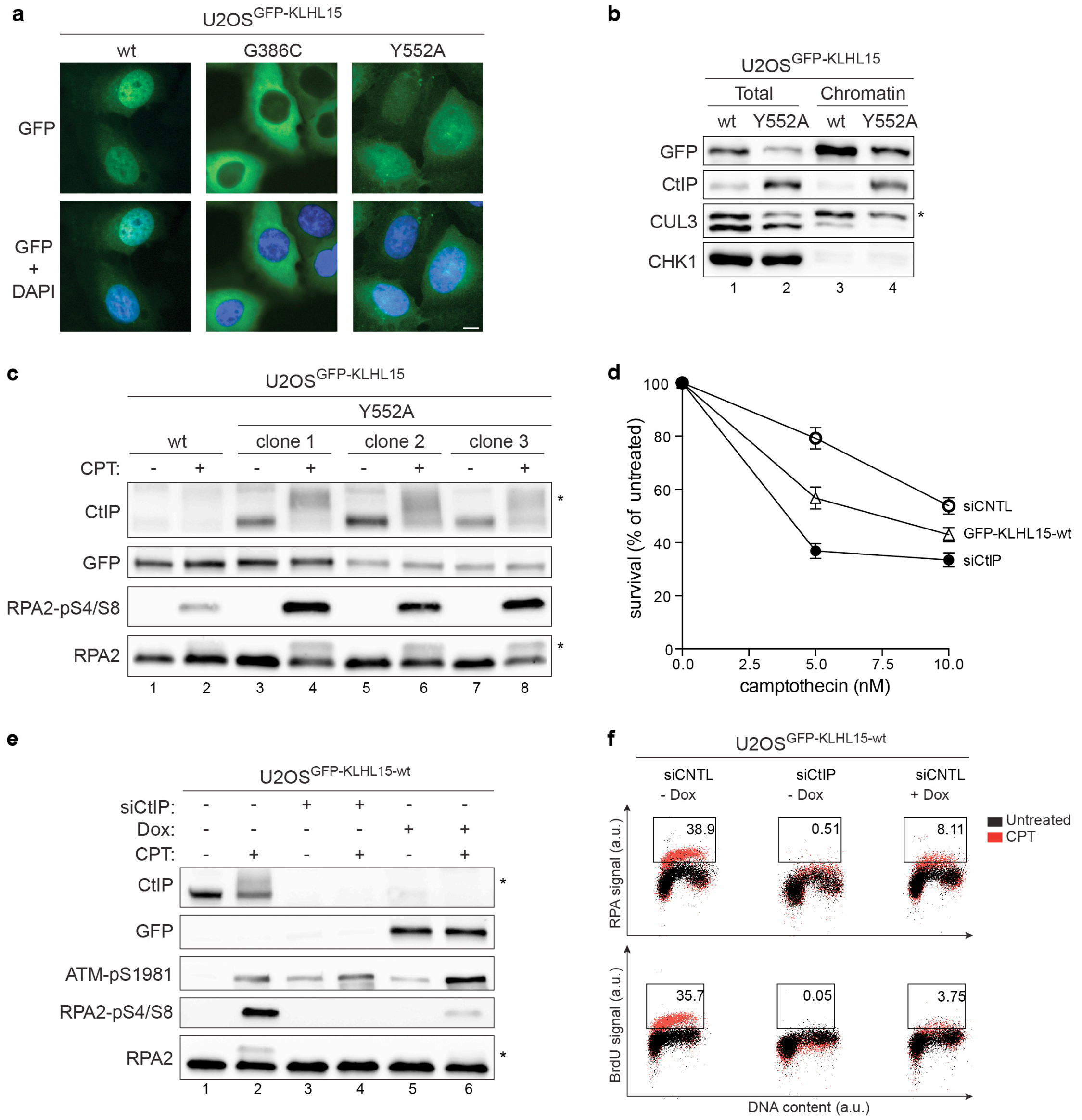

Supplementary Figure 4. Characterization of U2OS$^{\text{GFP-KLHL15}}$ cells. a, Immunofluroescence microscopy analysis of U2OS Flp-In T-REx cells inducibly expressing GFP-KLHL15-wt, GFP-KLHL15-G386C or GFP-KLHL15-Y552A cultivated for 24 h in presence of Dox. Scale bar, 10 µm. **b,** Total cell extracts and chromatin fractions of U2OS$^{\text{GFP-KLHL15-wt}}$ or U2OS$^{\text{GFP-KLHL15-Y552A}}$ cells were analysed by immunoblotting. Asterisk indicates neddylated CUL3. **c,** U2OS$^{\text{GFP-KLHL15-wt}}$ cells and three different single cell clones of U2OS$^{\text{GFP-KLHL15-Y552A}}$ were cultivated in presence of Dox. 24 h later, cells were mock-treated or treated with camptothecin (CPT, 1 µM) for 1 h and lysates were analysed by immunoblotting using the indicated antibodies. Asterisks indicate hyperphosphorylated CtIP and RPA2, respectively. **d,** U2OS$^{\text{GFP-KLHL15-wt}}$ cells were transfected with either non-targeting (CNTL) or CtIP siRNA oligos. 24 h later, KLHL15 expression in siCNTL-transfected cells was induced with Dox. 48 h post-siRNA transfection, cells were treated with the indicated doses of camptothecin. Survival was determined after four days using the CellTiter-Blue® cell viability assay. Data are presented as the mean ± SEM (n=3). **e**, Same cells as in **d** were mock-treated or treated with camptothecin (CPT, 1 µM) for 1 h and lysates were analysed by immunoblotting using the indicated antibodies. Asterisks indicate hyperphosphorylated CtIP and RPA2, respectively. **f**, Same cells as in **d** were labeled with BrdU (30 µM) for 24 h before treatment with CPT (1 µM) for 1 h. Cells were harvested, permeabilized, fixed, immunostained with anti-RPA2 or anti-BrdU antibody and analysed by FACS. Dot plots represent the intensity of the signals for RPA2 or BrdU staining (y axis) against the DNA content (x axis). Quantification gates were established in untreated samples and the percentage of cells within the gates are indicated. A.u., arbitrary units.

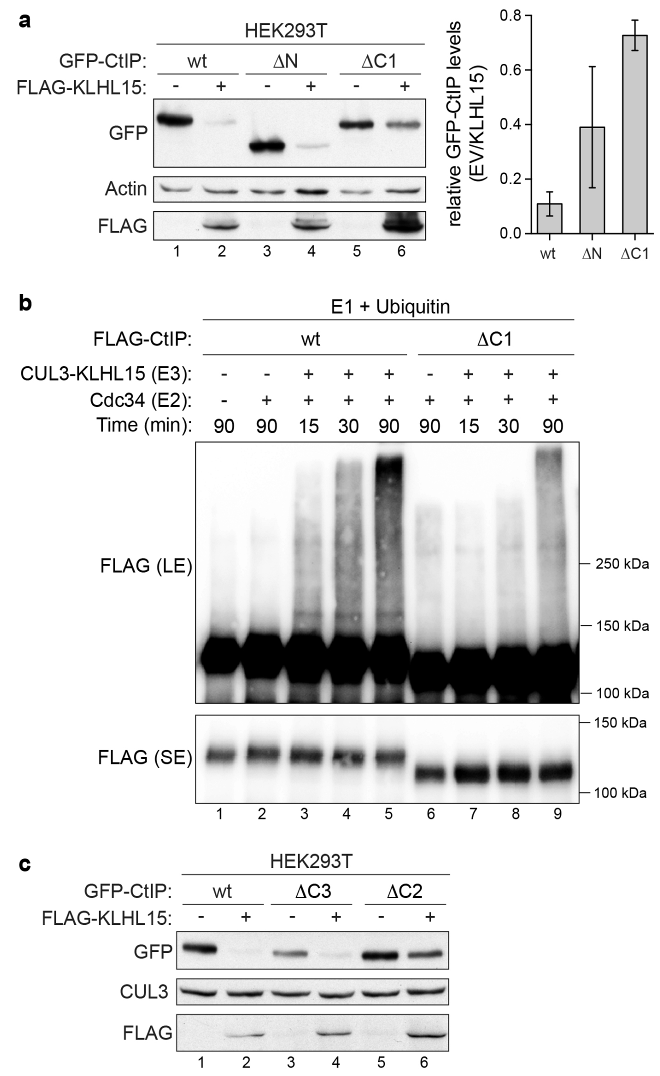

Supplementary Figure 5. KLHL15 binds to a region in the CtIP C-terminus. **a**, HEK293T cells were cotransfected with the indicated GFP-CtIP expression constructs together with either pcDNA3 empty vector (-) or FLAG-KLHL15. 48 h post-transfection, cells were analysed by immunoblotting (left panel). Relative CtIP protein levels were determined by quantification of CtIP band intensity (normalized to Actin) with the ImageJ software and expressed as ratios of the CtIP level in EV *versus* FLAG-KLHL15 transfected cells (right panel). Data are presented as mean values of densitometric quantification ± SEM (n ≥3). **b**, Recombinant FLAG-CtIP-wt or ΔC1 were incubated with ATP, ubiquitin, E1, Cdc34 and CUL3-RBX1-KLHL15 for the indicated time points at 37°C. Unmodified and ubiquitinated CtIP protein species were detected by immunoblotting with anti-FLAG antibody. SE and LE; short and long exposure times of the same immunoblot. **c**, HEK293T cells were cotransfected with the indicated GFP-CtIP expression constructs and FLAG-KLHL15. 48 h post-transfection, cells were analysed by immunoblotting.

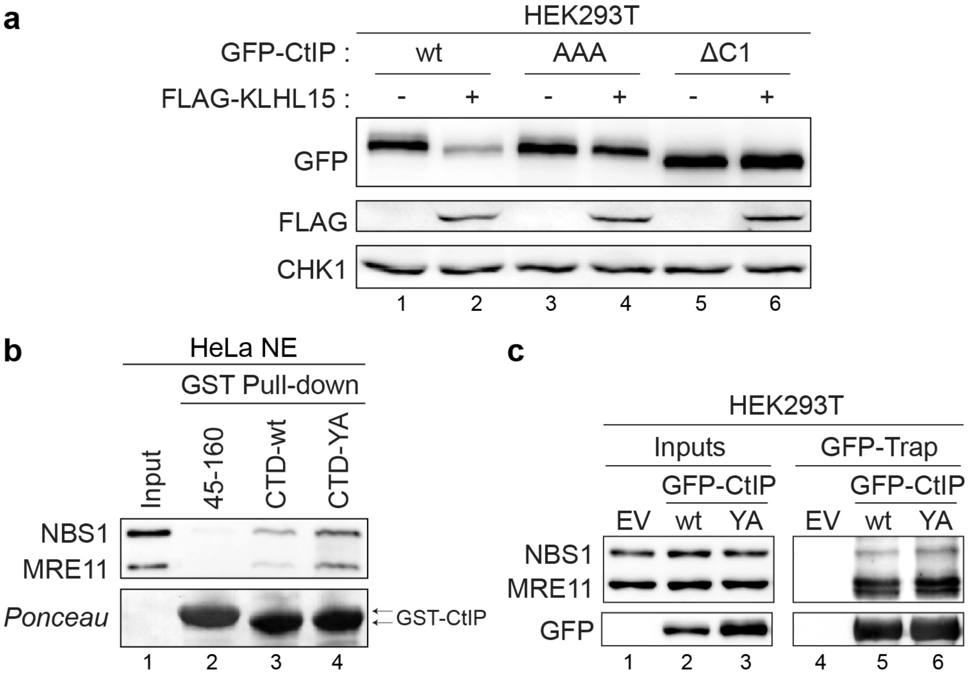

Supplementary Figure 6. A conserved 'FRY' motif in the CtIP C-terminal domain is responsible for its binding to KLHL15, but not for its interaction with MRE11 and NBS1. a, HEK293T cells were cotransfected with the indicated GFP-CtIP expression constructs and FLAG-KLHL15. 48 h post-transfection, cells were analysed by immunoblotting. **b**, Bacterially expressed GST fusion proteins of the CtIP coiled-coil domain (aa 45-160) and of the CtIP C-terminal domain (CTD, aa 790-897), either wt or Y842A (YA), were coupled to glutathione sepharose beads and incubated with 2 mg of HeLa nuclear extract (NE) for 1 h at 4C. Inputs and pulled-down were analysed by immunoblotting. **c**, HEK293T cells were transfected with either empty vector (EV) or the indicated GFP-CtIP expression constructs. 48 h post transfection, cells were lysed and whole-cell extracts were analysed by immunoblotting before (Input) or after immunoprecipitation using GFP-Trap beads.

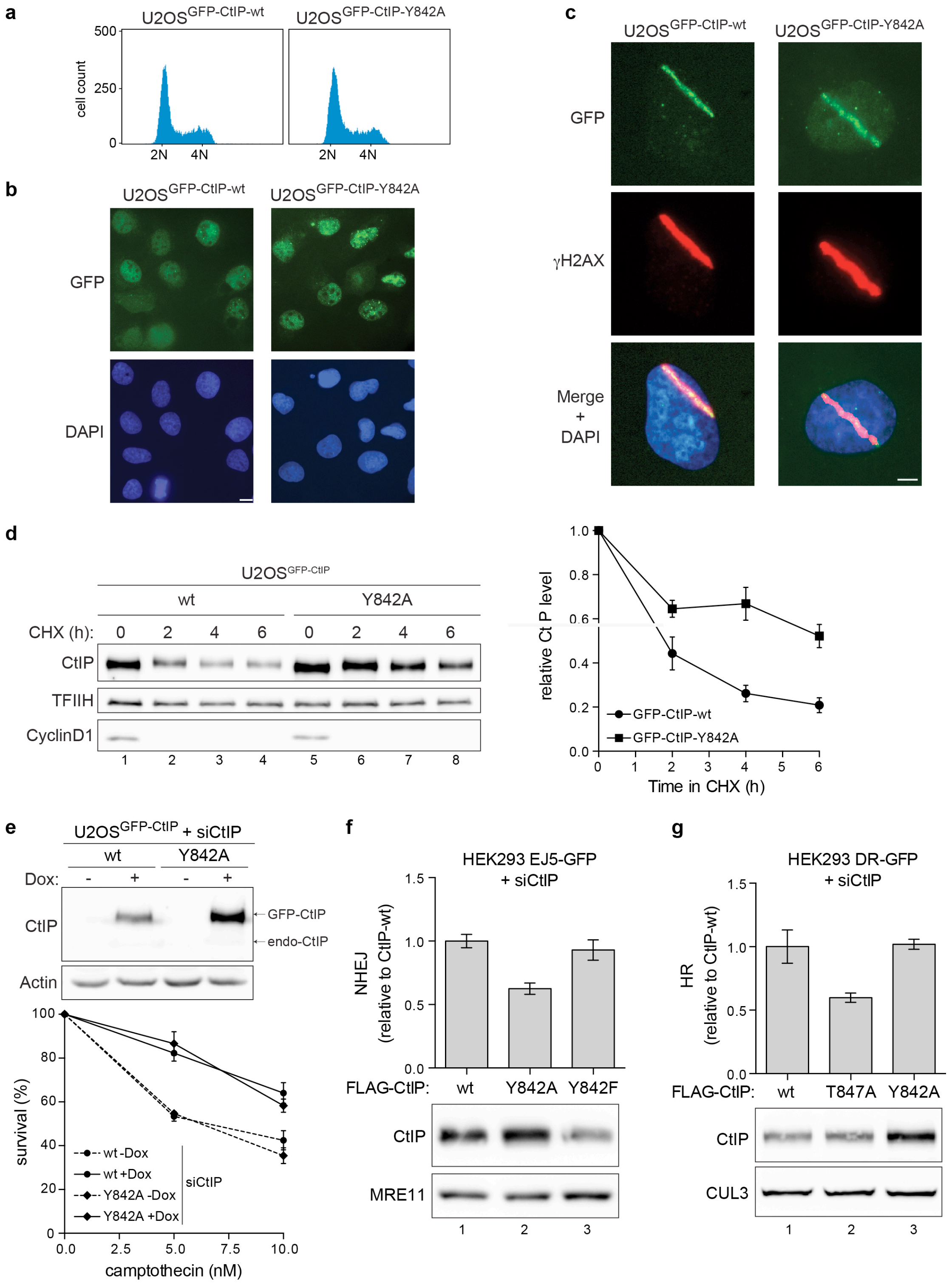

Supplementary Figure 7. Characterization of cells expressing CtIP-Y842A mutant defective in KLHL15 interaction. **a**, FACS analysis of U2OS Flp-In T-REx cells stably expressing doxycycline (Dox)-inducible siRNA-resistant GFP-CtIP-wt or GFP-CtIP-Y842A transfected with CtIP siRNA for 48 h and cultivated in the presence of Dox for the last 24 h. **b**, Same cells as in **a** were fixed and analysed by immunofluorescence microscopy. Nuclei were visualised by DAPI-staining. Scale bar, 10 µm. **c**, Same cells as in **a** were transfected with the CtIP siRNA for 48 h and cultivated in the presence of Dox for the last 24 h. 24 h after siRNA transfection, cells were grown on coverslips and sensitized with BrdU (10 µM) for 24 h prior to laser microirradiation. 20 min post-irradiation, cells were fixed, immunostained for γ-H2AX and analysed by fluorescence microscopy. Scale bar, 5 µm. **d**, Same cells as in **a** were treated with cycloheximide (CHX, 200 µg ml^{-1}) for the indicated time points and analysed by immunoblotting (left panel). Relative CtIP protein levels were determined by quantification of CtIP

band intensity (normalized to TFIIH) with the ImageJ software (right panel). Data are presented as mean values of densitometric quantification ± SEM (n =3). **e**, Same cells as in **a** were transfected with CtIP siRNA. 24 h post-transfection, cells were cultivated in absence or presence of Dox. 48 h post-transfection, cells were either lysed and analysed by immunoblotting (upper panel) or treated with the indicated doses of camptothecin and survival was determined after four days using the CellTiter-Blue® cell viability assay (lower panel). Data are presented as the mean ± SEM (n=3). **f,** HEK293 EJ5-GFP cells were transfected with CtIP siRNA. Two days later, cells were cotransfected with *I-SceI* in combination with the indicated siRNA-resistant FLAG-CtIP expression plasmids and harvested after 48 h for flow cytometry and immunoblot analysis. Data are represented as mean ± SEM (n=2). **g,** HEK293 DR-GFP cells were transfected with the CtIP siRNA. Two days later, cells were cotransfected with *I-SceI* in combination with the indicated siRNA-resistant FLAG-CtIP expression plasmids and harvested after 48 h for flow cytometry and immunoblot analysis. Data are represented as mean ± SEM (n=3).

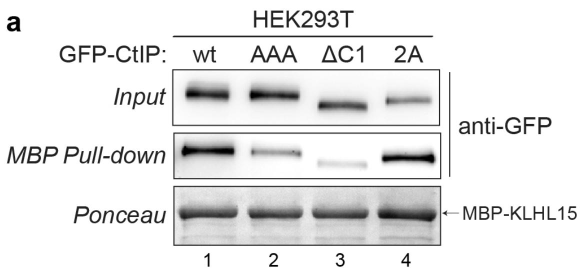

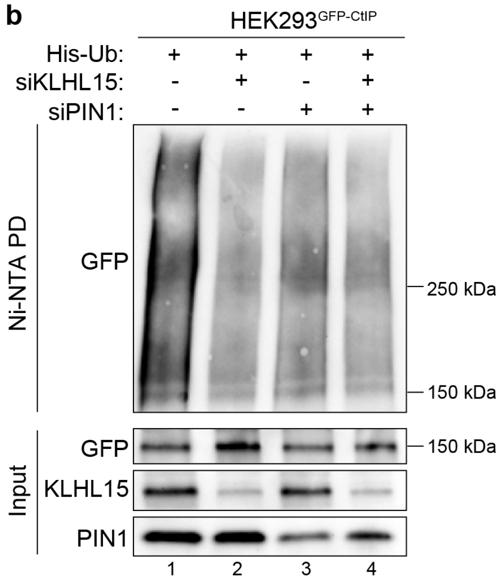

Supplementary Figure 8. PIN1 and KLHL15 collaborate in promoting CtIP ubiquitination. a, MBP-KLHL15 was coupled to amylose beads and incubated with lysates of HEK293T cells transfected with the indicated GFP-CtIP expression constructs for 48 h. Inputs and pulled-down protein complexes were analysed by immunoblotting. AAA, FRY (aa 840-842) residues, required for KLHL15 binding were substituted for alanines. 2A, S276 and T315 residues, requited for phosphorylation-dependent PIN1 binding, were substituted for alanines. **b,** HEK293 Flp-In T-REx cells inducibly expressing GFP-CtIP were transfected with the indicated siRNA oligos. 24 h later, cells were transfected with His-Ub and the expression of CtIP was simultaneously induced with Dox. Two days after siRNA transfection, cells were treated with MG-132 (20 µM) for 6 h followed by lysis in buffer containing guanidium-HCl. Ubiquitin conjugates were pulled-down (PD) with Ni-NTA-agarose beads, eluted and analysed by immunoblotting with anti-GFP antibody.

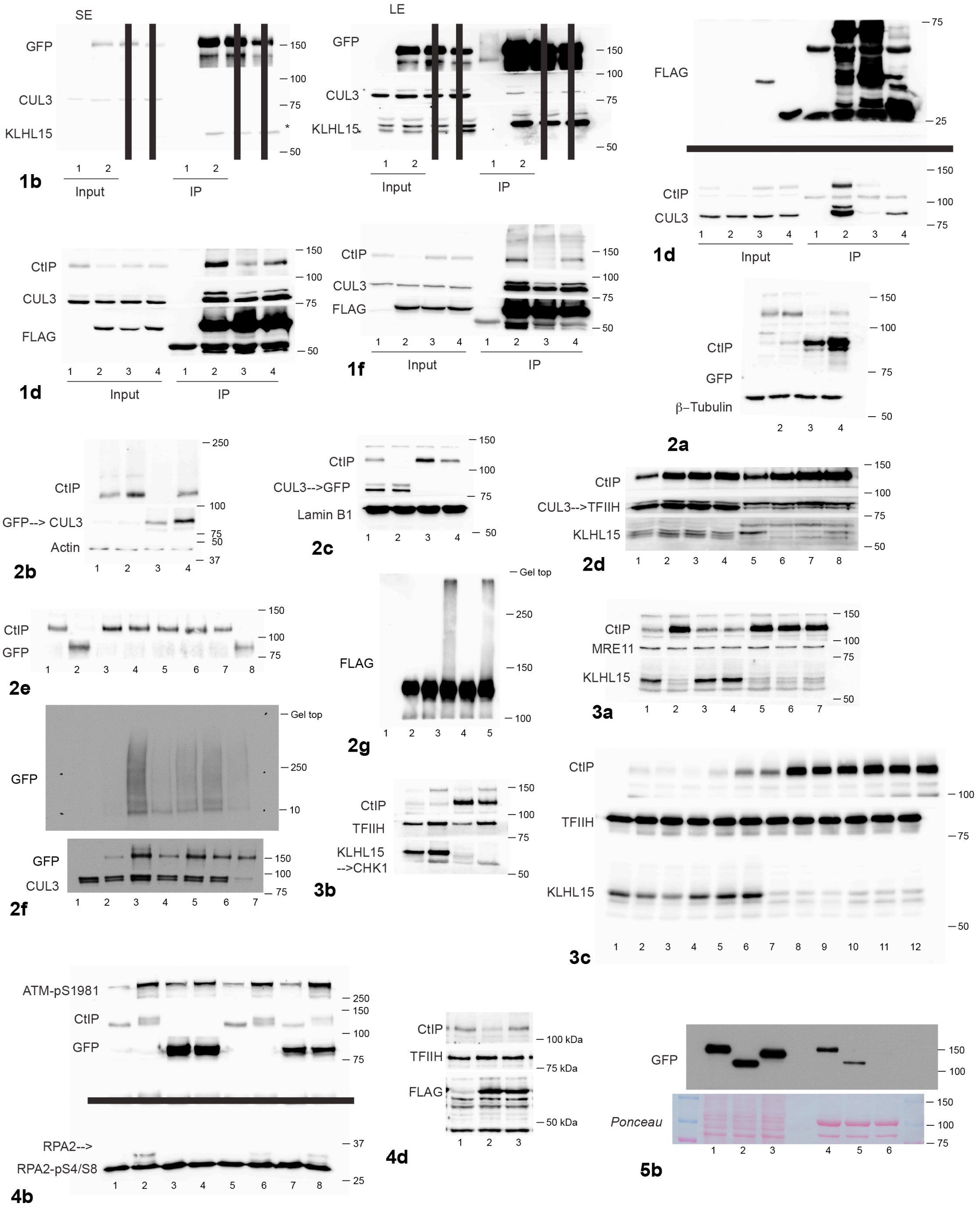

Supplementary Figure 9. Uncropped immunoblots. Labeling below each panel refers to the corresponding figure in the main article.

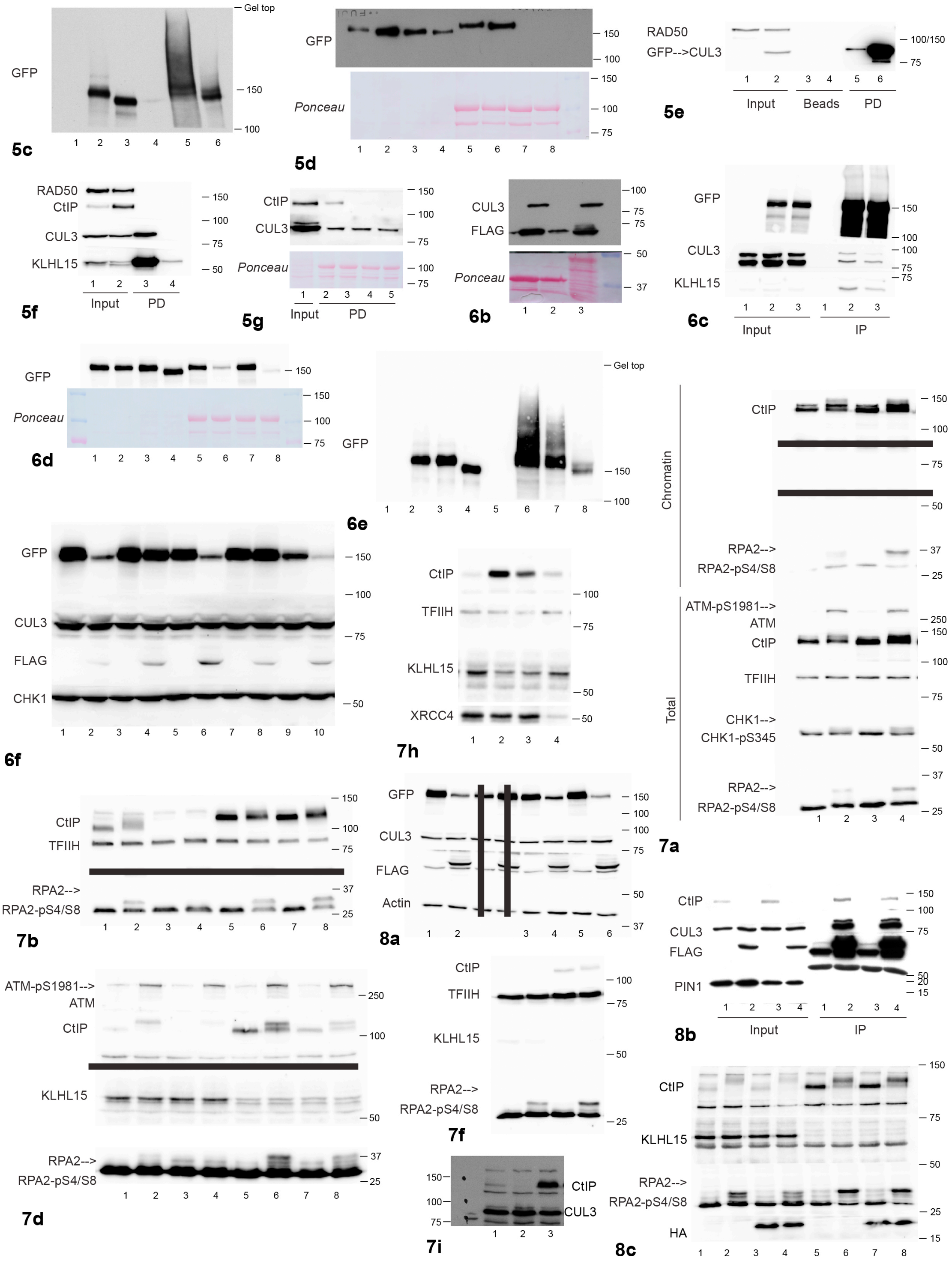

Supplementary Figure 9. Uncropped immunoblots. Labeling below each panel refers to the corresponding figure in the main article.

Antibody (Clone)	Host	Cat. No. (Company)	Applications (Dilutions)
ATM (2C1)	mouse	GT70103 (GeneTex)	WB (1:1000)
ATM-pS1981	rabbit	2152-1 (Epitomics)	WB (1:1000)
Actin (I-19)	rabbit	sc-1616 (Santa Cruz)	WB (1:1000)
β-Tubulin (D-10)	mouse	sc-5274 (Santa Cruz)	WB (1:20000)
BrdU	mouse	GERPN202 (GE Healthcare)	FACS (1:100)
CHK1 (G-4)	mouse	sc-8408 (Santa Cruz)	WB (1:100)
CHK1-pS345	rabbit	2341 (Cell Signaling)	WB (1:1000)
Cyclin A (H-432)	rabbit	sc-751 (Santa Cruz)	WB (1:1000)
Cyclin D1	rabbit	MS-210 (NeoMarkers)	WB (1:1000)
CtIP (14-1)	mouse	6114 (Active Motif)	WB (1:200)
CtIP (D-4)	mouse	sc-271339 (Santa Cruz)	WB (1:250)
CUL3	rabbit	A301-108A (Bethyl)	WB (1:2000)
FLAG	mouse	F3165 (Sigma)	WB (1:1000 - 1:10000)
GFP (B-2)	mouse	sc-9996 (Santa Cruz)	WB (1:100 - 1:5000)
HA (F-7)	mouse	sc-7392 (Santa Cruz)	WB (1:1000 - 1:10000)
H2AX-pS139 (20E3)	rabbit	9718 (Cell Signaling)	IF (1:500)
KLHL15	rabbit	*this study*	WB (1:500)
Lamin B1	rabbit	ab16048 (Abcam)	WB (1:1000)
MRE11 (12D7)	mouse	GTX70212 (GeneTex)	WB (1:1000)
NBS1 (1D7)	mouse	GTX70224 (GeneTex)	WB (1:1000)
PIN1	rabbit	2136-1 (Epitomics)	WB (1:1000)
RAD50 (13B3)	mouse	GTX70228 (GeneTex)	WB (1:1000)
RPA2 (RPA34-20)	mouse	NA19L (Calbiochem)	WB (1:1000); FACS (1:100)
RPA2-pS4/S8	rabbit	A300-245A (Bethyl)	WB (1:5000)
TFIIH p89 (S-19)	rabbit	sc-293 (Santa Cruz)	WB (1:1000)
Ubiquitin (P4D1)	mouse	sc-8017 (Santa Cruz)	WB (1:1000)
XRCC4	rabbit	GTX70263 (GeneTex)	WB (1:1000)

Supplementary Table 1: Primary antibodies. WB: Western blot, IF: Immunofluorescence Microscopy, FACS: Fluorescence-activated cell sorting.

Supplementary References

1. Zhuang, M. *et al.* Structures of SPOP-substrate complexes: insights into molecular architectures of BTB-Cul3 ubiquitin ligases. *Mol Cell* **36,** 39–50 (2009).
2. Lo, S.-C., Li, X., Henzl, M. T., Beamer, L. J. & Hannink, M. Structure of the Keap1:Nrf2 interface provides mechanistic insight into Nrf2 signaling. *The EMBO Journal* **25,** 3605–3617 (2006).
